# The Novel Nutraceutical KJS018A Prevents Hepatocarcinogenesis Promoted by Inflammation

**DOI:** 10.1155/2018/3909434

**Published:** 2018-08-01

**Authors:** Do Luong Huynh, Nisansala Chandimali, Jiao Jiao Zhang, Nameun Kim, Yang Ho Park, Taeho Kwon, Dong Kee Jeong

**Affiliations:** ^1^Laboratory of Animal Genetic Engineering and Stem Cell Biology, Advanced Convergence Technology & Science, Jeju National University, Jeju 63243, Republic of Korea; ^2^BRM Institute, Seoul 01756, Republic of Korea; ^3^Laboratory of Animal Genetic Engineering and Stem Cell Biology, Subtropical/Tropical Organism Gene Bank, Jeju National University, Jeju 63243, Republic of Korea

## Abstract

Inflammation is tightly associated with carcinogenesis at both the initiation and development of tumor. Many reports indicated that Cox-2 substantially contributes to inflammation and tumorigenesis. The novel nutraceutical KJS018A (BRM270 Function Enhanced Products) is the extract mixture from 8 herbal plants, which have been used to inhibit cancers and inflammation. The aim of the present study is to examine the inhibitory effects of KJS018A mixture to hepatocarcinogenesis and inflammation. The results showed that KJS018A significantly inhibited the proliferation of hepatic malignant cells and downregulated levels of IL-6 and Cox-2. Furthermore, KJS018A diminished the effect of PMA, an inflammatory inducer via IL-6/STAT3/Cox-2 pathway. Furthermore, KJS018A suppressed metastatic traits of hepatic malignant cells via downregulating Twist, N-cadherin, and MMP-9 while restoring E-cadherin expression. KJS018A also restrained tumor growth and levels of IL-6 and Cox-2 in immunohistochemistry staining. Taken together, these data suggest potential application of KJS018A in prevention of hepatocarcinogenesis promoted by inflammation.

## 1. Introduction

Several previous studies have shown that inflammation is strongly associated with carcinogenesis [1, 2]. Inflammation is involved in tumor development and is self-regulatory. It promotes the release of inflammatory inducers such as reactive oxygen species (ROS) and reactive nitrogen intermediates (RNIs), which in turn promote mutations [1]. Inflammation is also considered to activate cytokines secretion from white blood cells, including interleukin-6 (IL-6), interleukin 1 beta (IL-1*β*), and tumor necrosis factor-alpha (TNF-*α*). These cytokines promote proliferation, invasion, and angiogenesis of premalignant and tumor cells [1].

IL-6 is not only necessary for liver regeneration, but also crucial for tumorigenesis in liver [3]. IL-6 activates signal transducer and activator of transcription 3 (STAT3) signaling, resulting in Cox-2 expression [4, 5]. Cox-2 is associated with carcinogenesis, and its presence has been found to be upregulated in various types of cancers in both animal models or clinical cancers [6, 7]. These evidences led to a conclusion that Cox-2 contributes to the hallmarks of cancer, including evasion of apoptosis, self-maintaining cell survival and proliferation, sustaining angiogenesis, promoting tissue invasion, and metastasis [6]. Therefore, inhibition of Cox-2 might be an effective approach to disrupt inflammation-hepatocarcinogenesis interlink.

KJS018A (BRM270 Function Enhanced Products) is herbal extract from 8 plants including* Aloe vera*,* Arnebia euchroma*,* Citrus unshiu Markovich*,* Portulaca oleracea*,* P. vulgaris var. lilacina, Saururus chinensis, Scutellaria baicalensis,* and* Piper longum* which are believed to prevent tumorigenesis and inflammation [8–15]. In this study, we aim to explore the effects of KJS018A to inflammation-hepatotumorigenesis interlink and results showed that IL-6 and Cox-2, which are inflammation and tumorigenesis markers, were downregulated under KJS018A treatment. Moreover, KJS018A suppressed STAT3 phosphorylation at tyrosine 705 site and subsequently decreased either STAT3 downstream target genes or metastatic phenotype in malignant cells. These evidences provide a new insight of KJS018A application in antihepatocarcinogenesis and might help confine the contributions of IL-6 and Cox-2 to cancer development.

## 2. Materials and Methods

### 2.1. Reagents

KJS018A supplied by BRM institute, Seoul, Republic of Korea, was extracted using methanol/ethanol followed by rotary concentration. The KJS018A pellet was dissolved in Dimethyl Sulfoxide (DMSO; Sigma-Aldrich, USA) and stored at −20°C for further analysis. Phorbol myristate acetate (PMA) and polyclonal antibodies against Bcl-2, Bax, caspase-3, IL-6, E-cadherin, twist, and matrix metallopeptidase-9 (MMP-9) antibodies and horseradish peroxidase- (HRP-) conjugated anti-rabbit or anti-mouse immunoglobulin G (IgG) were purchased from Santa Cruz Biotechnology (Santa Cruz, California, USA). Akt, pAkt, Cox-2, ERK1/2, pERK1/2, GAPDH, p53, N-cadherin, STAT3, and pSTAT3 Y705 were purchased from Abfrontier (Seoul, Republic of Korea).

### 2.2. Cell Culture

SNU398 and HepG2 cells were cultured in RPMI and DMEM, respectively, supplemented with 10% fetal bovine serum (FBS) and 1% antimycotic. Cultures were maintained in an incubator (5% CO_2_, 37°C).

### 2.3. Cell Viability

A total of 5 × 10^3^ cells was seeded into a 96-well plate and incubated for 24 h before treatment with various concentrations of KJS018A (0.01, 0.1, 1, 10, 100, and 1000 *μ*g/mL). After 24 h, cell viability was measured using an EZ-cytox kit (Daeil lab service, Seoul, Korea) according to the manufacturer's protocol. The cell viability test results were presented as the ratio of optical density at 450 nm (OD_450 nm_), which was calculated using the following formula: (%) cell viability = (OD treatment groups or control groups/OD vehicle control group) × 100%.

### 2.4. Annexin V Apoptosis Assay

A number of 1 × 10^5^ cells after 24 h exposed to various concentrations of KJS018A (0, 50, 100, and 150 *μ*g/mL) were performed apoptosis assay according to the manufacturer's protocol (BD Biosciences, NJ, US). In brief, harvested cells were washed with Annexin V binding buffer and resuspended in 100 *μ*L binding buffer containing 5 *μ*L FITC-conjugated Annexin V and 5 *μ*L propidium iodide for exactly 15 min in the dark at room temperature (RT-25°C). Cells were then analyzed using a BD Accuri C6 cytometer (BD Biosciences, NJ, US).

### 2.5. Wound Healing Assay

Wound healing assay was carried out according to the manufacturer's protocol (Essen Bioscience, MI, US). In brief, 5 × 10^4^ cells in the log phase were seeded into each well of 96-well plate overnight, to reach 98–100% confluence. A monolayer of cells was scratched by using a wound maker, supplemented with 0.5% FBS medium containing 50 *μ*g/mL KJS010A and imaged real-time by IncuCyte system (Essen Bioscience, MI, US).

### 2.6. In Vitro Cell Migration and Invasion Assays

Cell migration assay was performed by using 8-*μ*m pore size cell culture inserts (Merck Millipore, MA, USA). Briefly, the lower chamber was filled with 0.8 mL of complete DMEM with 20% FBS. Cells, pretreated with KJS018A, were harvested by using trypsin/EDTA and washed twice with serum-free DMEM. Subsequently, 1 × 10^5^ cells in 0.2 mL of migration medium (complete DMEM with 0.5% FBS) were added to the upper chamber. After incubation for 48 h at 37°C, cells on the upper surface of the membrane were removed by cotton swabs. The migrating cells were fixed in 3.7% paraformaldehyde (PFA) for 10 min. Next, cells were treated with prechilled absolute methanol at room temperature (RT-25°C) for 20 min and stained with a solution containing 0.5% crystal violet for 30 min. Migrating cell number was counted by visualizing on microscope in five random fields at 100 ×. For the cell invasion assay, all the procedures were carried out as the migration assay, except coating matrigel matrix growth factor-reduced basement membrane (BD Biosciences, NJ, US) (3.5 mg/mL) on the upper and followed by incubation at 37°C before adding cells to the upper chamber.

### 2.7. Western Blotting

A total of 1 × 10^6^ cells exposed with different concentrations of KJS018A (0, 50, 100, and 150 *μ*g/mL) were collected and lysed for 30 min in ice-cold RIPA (radioimmunoprecipitation assay) buffer supplemented with phenylmethylsulfonyl fluoride (PMSF), protease inhibitor cocktail, and sodium orthovanadate according to the manufacturer's protocol. After removing cell debris by centrifugation at 10,000 ×*g* for 15 min, equal amounts of proteins were separated by 12% SDS-PAGE, transferred electrophoretically (Bio-Rad, CA, USA) onto a polyvinylidene fluoride (PVDF) membrane, and blocked with 5% nonfat milk powder (w/v) in phosphate-buffered saline plus 0.1% Tween-20 (PBST 1X) for 1 h at room temperature (RT- 25°C). The membranes were incubated overnight at 4°C with primary antibodies or with anti-GAPDH mouse monoclonal antibody as an internal control. After washing five times with PBST 1X,* horseradish peroxidase-* (HRP-) conjugated anti-rabbit or anti-mouse secondary antibodies were added and incubated at 37°C for 2 h before another five-time washing. The bands were captured using ImageQuant™ LAS 4000 mini Fujifilm.

### 2.8. Immunocytochemistry Staining

Cells treated with 50 *μ*g/mL KJS018A or 0.1% DMSO were fixed by using 3.7% PFA. Before overnight staining with primary antibodies of interest, cells were blocked with PBST 1X plus 3% Bovine Serum Albumin (BSA). After washing two times with PBST 1X, secondary antibodies were added and followed by 2 h incubation. Cells next were stained for 10 min with 4′,6-diamidino-2-phenylindole (DAPI) before visualizing on microscopic images.

### 2.9. Xenograft Tumor Model

Male nude mice were randomly divided into two groups and 5 × 10^6^ cells were injected subcutaneously at flank of mice. One week after injection, tumors were measured by calipers weekly. All mice were daily oral uptake with PBS or KJS018A 5 mg/kg. After 5 weeks for inoculation, mice were sacrificed to harvest tumors. Tumor volumes were calculated by the formula: *π* x (Length x Width x Height)/6. Tumors were fixed in 4% paraformaldehyde (PFA) and embedded in paraffin before immunohistochemistry staining (IHC) analysis.

### 2.10. Immunofluorescent Staining

Tissues fixed in 4% PFA and paraffin-embedded were sliced into 4 *μ*m sections, followed by deparaffinization in xylene, rehydration in graded ethanol solutions, and 3% hydrogen peroxide/methanol solution for 30 min. After 3 times washed by PBS Triton X-100 (0.5%), tumor slices were incubated with blocking solution (5% sheep serum) in 30 min and followed by primary antibody overnight incubation in moisture chamber. On the next day, tumor slices were incubated 2 h in secondary antibody-conjugated with phycoerythrin (PE) in the dark and stained with DAPI 20 min/RT. After 3 washing, tumor slices were covered by mounting solution slip and checked on inverted fluorescence microscope.

### 2.11. Statistical Analysis

Statistical analysis was performed using Graphpad Prism 6.02. Data were expressed as mean ± standard deviation (SD). Experimental differences were examined using ANOVA and Student's* t*-tests, as appropriate.* P *values of <0.05 were considered to indicate significantly statistical difference.

## 3. Results

### 3.1. KJS018A Induces Apoptosis in Hepatic Malignant Cells

**Figure 1:**
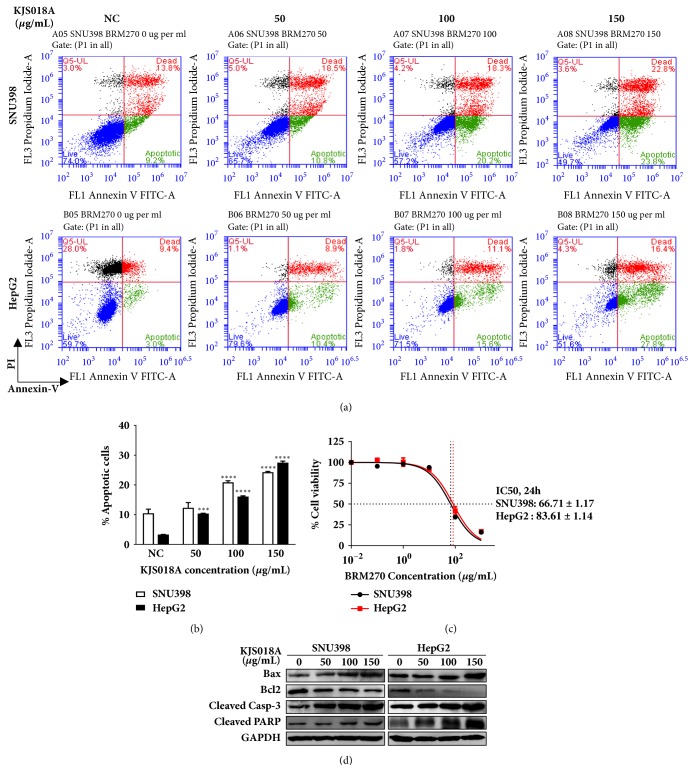
*KJS018A induces apoptosis in hepatic malignant cells.* (a) Apoptosis assay of SNU398 and HepG2 after being treated with different concentrations of KJS018A (0, 50, 100, and 150 *μ*g/mL). (b) Percentage of apoptotic cells after KJS018A treatment (0, 50, 100, and 150 *μ*g/mL). (c) IC_50_ at 24 h for SNU398 and HepG2 under KJS018A treatment. (d) Western blotting of SNU398 and HepG2 lysates after 24 h exposed with different concentrations of KJS018A (0, 50, 100, and 150 *μ*g/mL). ^*∗∗∗*^*P< 0.001; *^*∗∗∗∗*^*P< 0.0001. *The experiment was repeated 3 times.

Cells exposed to various concentrations of KJS018A (0, 50, 100, and 150 *μ*g/mL) were subjected to FACS apoptosis assay using Annexin V-FITC and western blotting. As shown in Figures 1(a) and 1(b), the apoptotic percentage of hepatic malignant cells under KJS018A expose were increased in a dose-dependent manner. The cell viability assay showed that the IC_50_ values of SNU398 and HepG2 were 66.71 *μ*g/mL and 83.61 *μ*g/mL, respectively (Figure 1(c)). Western blotting data indicated either the activation of caspase-3 and PARP while decreasing Bcl2 in hepatic malignant cells (Figure 1(d)). Furthermore, KJS018A also upregulated Bax level, leading to apoptosis via intrinsic pathway (Figure 1(d)). These data suggested that KJS018A treatment is able to inhibit the proliferation of hepatic malignant cells by inducing apoptosis.

### 3.2. KJS018A Prevents the Expressions of PMA-Induced Inflammatory Markers via Suppressing STAT3 Signaling Pathway

**Figure 2:**
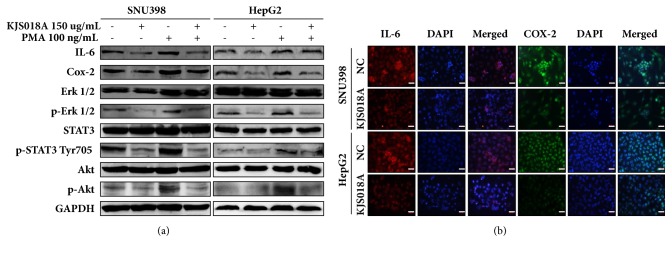
*KJS018A suppresses PMA-promoted inflammation in hepatic malignant cells via inhibiting Cox-2/IL-6 axis.* (a) Western blotting of SNU398 and HepG2 after 150 *μ*g/mL KJS018A treatment for 24 h with or without 100 ng/mL PMA activation. (b) Attenuation of IL-6 and Cox-2 in SNU398 and HepG2 after 24 h exposing 50 *μ*g/mL KJS018A, visualized by immunocytochemistry staining. Scale bar, 20 *μ*m.

Several studies have indicated that inflammation is highly involved in carcinogenesis [1, 2]. In this study, we established the phorbol-12-myristate-13-acetate (PMA) induced inflammation in hepatic malignant cells to examine whether KJS018A can prevent inflammation-promoted hepatocarcinogenesis. Indeed, previous reports have shown that PMA is a strong mediator of inflammation [16]. Furthermore, PMA is also considered a Cox-2 inducer. PMA increases Cox-2 expression, which promotes hepatocarcinogenesis progress and enhances the aggression of inflammation [6, 16, 17]. As shown in Figure 2(a), PMA upregulated the expressions of IL-6 and Cox-2 while activating ERK1/2, Akt, and STAT3 signaling pathways. In contrast, KJS018A downregulated levels of IL-6 and Cox-2 while inhibiting the phosphorylation of such signaling pathways and preventing aggressive inflammation from PMA treatment (Figure 2(a)). Moreover, results unveiled that KJS018A inhibited STAT3 signaling at tyrosine 705 site and repressed both levels of IL-6 and Cox-2 in immunocytochemistry staining (Figure 2(b)). Taken together, these data demonstrated that KJS018A intercepts PMA-mediated inflammation and inhibits hepatocarcinogenesis progress via suppressing IL-6/STAT3/Cox-2 axis.

### 3.3. KJS018A Inhibits Metastatic Traits in Hepatic Malignant Cells

**Figure 3:**
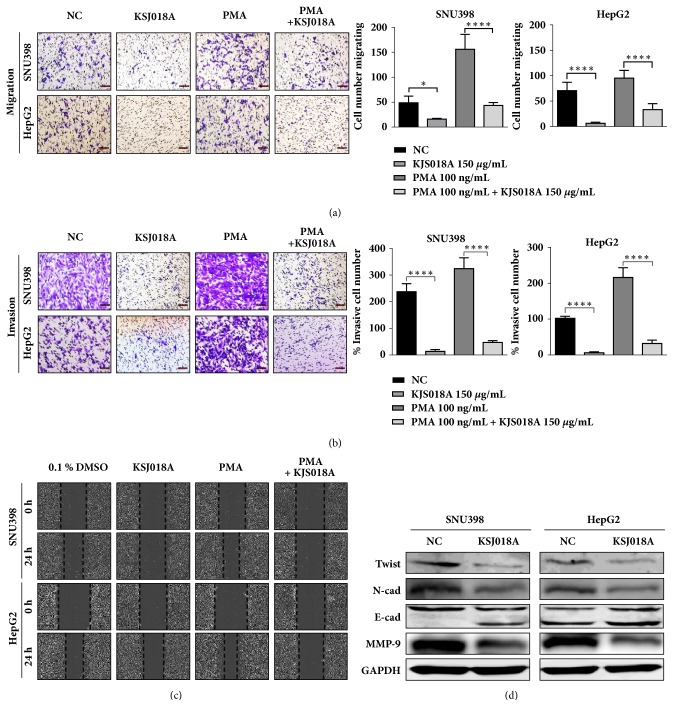
*KJS018A inhibits metastatic traits in hepatic malignant cells.* (a) Migration assay of SNU398 and HepG2 in presence of 50 *μ*g/mL KJS018A with or without 100 ng/mL PMA activation. (b) Invasion assay of SNU398 and HepG2 in presence of 50 *μ*g/mL KJS018A with or without 100 ng/mL PMA activation. (c) Wound healing assay of SNU398 and HepG2 treated with 50 *μ*g/mL KJS018A with or without 100 ng/mL PMA activation. (d) EMT markers in lysates of SNU398 and HepG2 after 24 h exposed with 50 *μ*g/mL KJS018A treatment. ^*∗*^*P< 0.05; *^*∗∗∗∗*^*P< 0.0001. *Scale bar, 50 *μ*m. The experiment was repeated 3 times.

Cox-2 expressions is also tightly associated with metastatic traits such as EMT, cell migration, and invasion [6, 18]. Overexpression of Cox-2 subsequently promotes metastasis rapidly and leads to poor patient prognosis [6]. Therefore, Cox-2 downregulation would potentially inhibit the mobility of malignant cells. In this study, our data showed that KJS018A inhibited Cox-2 expression; however, it is unclear whether this prevents hepatic malignant cells mobility. To address this question, hepatic malignant cells were exposed to KJS018A 24 h prior to migration and invasion assay. Number of cells migrating and invading were significantly decreased after exposed to KJS018A (Figures 3(a) and 3(b)). In addition, wound healing assay indicated that KJS018A considerably inhibited cell mobility at 50 *μ*g/mL (Figure 3(c)). However, PMA treatment was unable to promote cell mobility in presence of KJS018A (Figures 3(a), 3(b), and 3(c)). Further analysis showed that KJS018A targeted EMT protein markers: Twist, N-cadherin, and MMP-9 were downregulated, while restoring E-cadherin expression (Figure 3(d)). Taken together, these data indicated that KJS018A effectively minimizes the metastatic traits of hepatic malignant cells.

### 3.4. KJS018A Suppresses Hepatic Tumor Growth via Downregulation of IL-6 and Cox-2

**Figure 4:**
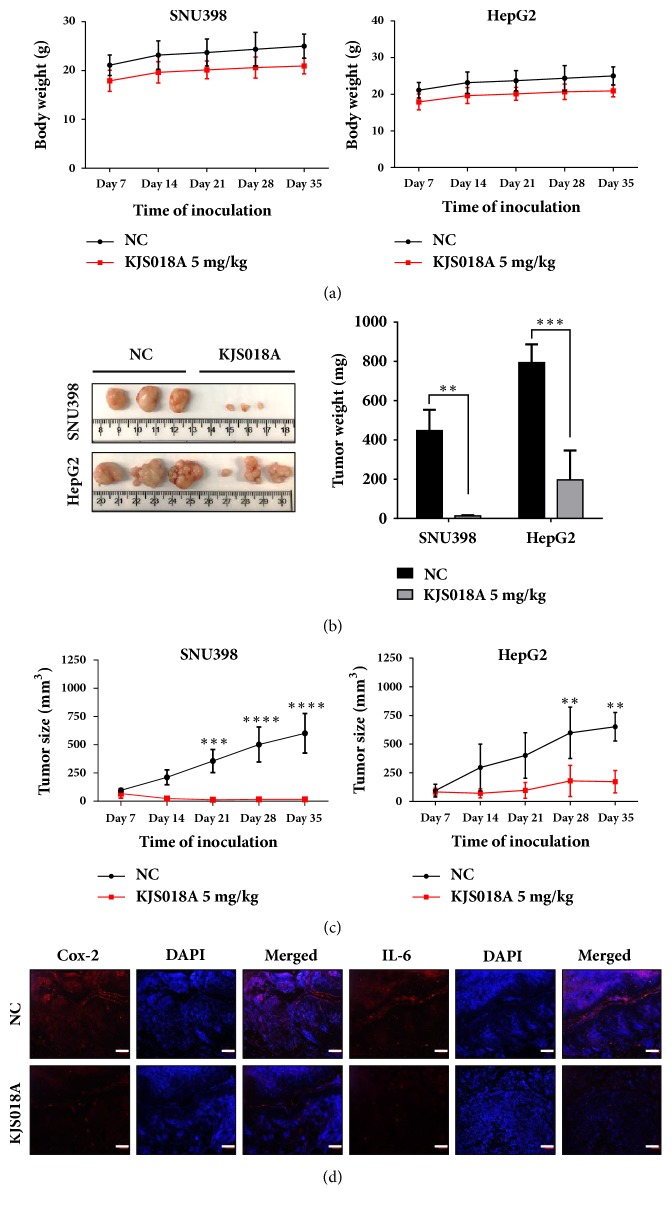
*KJS018A suppresses hepatic tumor development via downregulation of Cox-2 and IL-6.* (a) Body weights of mice with and without KJS018A treatment (5 mg/kg). (b) Tumor sections (left) and weights (right) with and without KJS018A treatment (5 mg/kg). (c) Tumor sizes of SNU398 and HepG2 with and without KJS018A treatment (5 mg/kg). (d) Immunofluorescence staining of Cox-2 and IL-6 expression in hepatic tumors with and without KJS018A treatment (5 mg/kg). Scale bar, 20 *μ*m. ^*∗∗*^*P< 0.01; *^*∗∗∗*^*P< 0.001; *^*∗∗∗∗*^*P< 0.0001.*

Clinical studies showed that the overexpression of IL-6 and Cox-2 contributes significantly to tumor growth [6, 19–21] and blockades of IL-6 and Cox-2 signaling are potential chemotherapies [17, 20–22]. Furthermore, our previous studies showed that BRM270, former version of KJS018A, enables suppressing tumor growth in lung adenocarcinoma [23, 24]. Therefore, in this study we investigated inhibitory effects of KJS018A to hepatic tumor growth via xenografting SNU398 and HepG2 hepatic malignant cells in nude mice. Results showed that mice body weights as compared to nontreatment were not significantly different (Figure 4(a)). KJS018A impaired considerably tumor growths of SNU398 and HepG2 and exhibited the reductions of tumor formations, weights (Figure 4(b)), and tumor sizes (Figure 4(c)). Furthermore, immunohistochemistry staining indicated that expressions of IL-6 and Cox-2 in tumors were attenuated by KJS018A treatment (Figure 4(d)). These results indicated that KJS018A prevents hepatocarcinogenesis via interrupting the contributions of IL-6 and Cox-2 to tumor growth.

## 4. Discussion and Conclusion

Previous studies have shown that inflammation is a key process during tumor development [1, 2]. Inflammation helps promote a series of cancer hallmarks, such as cell proliferation, survival, resistance, angiogenesis, and metastasis [2]. Increasing evidences indicated that Cox-2 is highly associated with inflammation in the tumor microenvironment [6]. One of the main signaling pathways triggering Cox-2 expression in malignant cells is IL-6/STAT3. This pathway is responsible for the intracellular inflammatory responses and indirectly induces Cox-2 overexpression, leading to poor patient prognosis [6].

Our previous study showed that BRM270 efficiently inhibits the activity of NF-*κ*B, resulting in the downregulation of CDK6 and IL-6 in osteosarcoma CSCs [23]. In this study, new advanced extract of BRM270, KJS018A, prevents the development of hepatic malignant cells by downregulating the activity of IL-6/STAT3, consequently inhibiting intracellular Cox-2 levels. KJS018A inhibits the phosphorylation of STAT3 at the tyrosine 707 site, whereas there were no changes at the serine 727 site (data not shown), leading to the suppression of downstream genes. These data are consistent with those of previous studies, which suggest that the tyrosine 707 site is mainly activated by IL-6/JAK, Src or RTK signaling, whereas the serine 727 site is phosphorylated by MAPK, mTOR, PKC, or IKK [25].

PMA is a well-known inflammatory activator [16]. PMA increases the levels of IL-6 by enhancing its promoter activity [26–29], which in turn activates STAT3 signaling, resulting in expression of downstream genes, including Cox-2 or EMT genes and inflammatory responses [3–5]. Results showed that KJS018A disables PMA contributions to hepatocarcinogenesis by interfering signaling pathways such as Akt, ERK1/2 or STAT3. Some studies have reported that PMA is responsible for the activation of signaling cascades such as NF*κ*B, PI3K/Akt/C-jun, MAPK, and ERK; however, there is lack of evidence regarding the activation of STAT3 by PMA [30–32]. These data also suggested that PMA activity stimulates PKC cascades, which in turn phosphorylate downstream cascades and genes, directly upregulating the expression of IL-6 and Cox-2 [32–34]. However, IL-6 acts as an autocrine ligand, which promotes STAT3 signaling via a feed-back mechanism and promotes inflammatory responses in the tumor microenvironment. Therefore, it can be concluded that STAT3 activation was indirectly induced by PMA. In this study, PMA mediates the expressions of IL-6 and Cox-2 while increases wound healing, migration, and invasion ability in hepatic malignant cells. These evidences demonstrate PMA contribution to hepatic cancer progression. However, KJS018A prevents efficiently the expressions of IL-6 or Cox-2 in presence of PMA.

In previous studies, we have optimized and figured out that 5 mg/kg BRM270 dose could inhibit efficiently hepatocellular carcinoma (HCC) tumor growths [23, 35, 36]. Thus, in this study we have examined new form KJS018A as the same dose of BRM270 and results show that KJS018A also unfavorites IL-6 and Cox-2* in vivo* while not affecting mice body weight. The dose of KJS018A might change depending on type of cancers and further studies for optimal dose or pharmacokinetics of KJS018A are necessary. Nevertheless, we believe that KJS018A prevents effectively hepatocarcinogenesis promoted by inflammation both* in vitro* and* in vivo*. Therefore, KJS018A could be a potential inhibitor to circumvent inflammation-tumorigenesis bond.

## Data Availability

The data used to support the findings of this study are available from the corresponding author upon request.
